# Transient *In Vivo* Resistance Mechanisms of *Burkholderia pseudomallei* to Ceftazidime and Molecular Markers for Monitoring Treatment Response

**DOI:** 10.1371/journal.pntd.0005209

**Published:** 2017-01-12

**Authors:** Jason E. Cummings, Richard A. Slayden

**Affiliations:** Department of Microbiology, Immunology and Pathology, Colorado State University, Fort Collins, Colorado, United States of America; Beijing Institute of Microbiology and Epidemiology, CHINA

## Abstract

Much is known about the mode of action of drugs and resistance mechanisms under laboratory growth conditions, but research on the bacterial transcriptional response to drug pressure *in vivo* or efficacious mode of action and transient resistance mechanisms of clinically employed drugs is limited. Accordingly, to assess active alternative metabolism and transient resistance mechanisms, and identify molecular markers of treatment response, the *in vivo* transcriptional response of *Burkholderia pseudomallei* 1026b to treatment with ceftazidime in infected lungs was compared to the *in vitro* bacterial response in the presence of drug. There were 1,688 transcriptionally active bacterial genes identified that were unique to *in vivo* treated conditions. Of the *in vivo* transcriptionally active bacterial genes, 591 (9.4% coding capacity) genes were differentially expressed by ceftazidime treatment. In contrast, only 186 genes (2.7% coding capacity) were differentially responsive to ceftazidime treatment under *in vitro* culturing conditions. Within the genes identified were alternative PBP proteins that may compensate for target inactivation and transient resistance mechanisms, such as β-lactamses that may influence the potency of ceftazidime. This disparate observation is consistent with the thought that the host environment significantly alters the bacterial metabolic response to drug exposure compared to the response observed under *in vitro* growth. Notably, this study revealed 184 bacterial genes and ORFs that were unique to *in vivo* ceftazidime treatment and thus provide candidate molecular markers for treatment response. This is the first report of the unique transcriptional response of *B*. *pseudomallei* from host tissues in an animal model of infection and elucidates the *in vivo* metabolic vulnerabilities, which is important in terms of defining the efficacious mode of action and transient resistance mechanisms of a frontline meliodosis chemotherapeutic, and biomarkers for monitoring treatment outcome.

## Author Summary

The mode of action and resistance mechanisms for many clinically used drugs are elucidated under defined artificial laboratory growth conditions, and do not necessarily represent the efficacious mode of action or predict the transient resistance mechanisms that are active during infection. Here, we report the unique transcriptional response of *B*. *pseudomallei* from host tissues and demonstrate the efficacious mode of action and transient resistance mechanisms of the frontline meliodosis chemotherapeutic, ceftazidime.

## Introduction

Understanding the mode of action and potential resistance mechanisms of frontline chemotherapeutics provides information about how clinically used drugs exert their efficacious effect. Typically the lethal effect of a drug is investigated in the laboratory under artificial conditions and is defined as the drugs mode of action [1]. Here we expand on this concept by assessing the bacterial transcriptional response, and thus metabolic response to drug treatment and transient resistance mechanisms *in vivo*, and use the term efficacious mode of action to distinguish between analyses performed using bacteria from *in vitro* or *in vivo* sources. An important implication is the laboratory-defined mode of action is often used to substantiate a bacterial protein or metabolic pathway for inhibitor design and to inform potential drug resistance mechanisms. The primary drawback of using *in vitro* only information is that the defined *in vitro* mode of action may not be the same as the efficacious *in vivo* mode of action or *in vivo* specific metabolism or mechanisms that influence susceptibility and resistance. This possibility results from the bacterial alternative metabolism or use of coding redundancy as a result of the host environment, which supersedes the pressure of drug exposure.

The well-documented intrinsic resistance of *Burkholderia* spp. to drug treatment is primarily attributed to the extensive efflux capability of the pathogen [2]. However, *Burkholderia* spp. are also known for extensive coding capacity, which provides alternative or redundantly encoded components or metabolic capabilities available to the bacteria during stress and treatment pressure [3]. Since an appreciation of the impact of coding redundancy on *in vivo* drug susceptibility is just emerging, there is a need to understand the metabolic activities of the bacteria under disease conditions in the host environment [3]. Further, to validate the therapeutic value of a putative drug target it is critical to evaluate essentiality during infection and disease dissemination in a host [3]. Throughout our own drug discovery program we have encountered conditional lethal drug targets, that differ significantly in their essentiality between *in vitro* and *in vivo* growth environments [3,4].

Ceftazidime, a third-generation β-lactam cephalosporin, is the front line standard of care therapeutic when treating acute melioidosis [2]. The mode of action of ceftazidime has been reported to be inhibition of the penicillin-binding protein 3 (PBP-3), FtsI, which when inhibited leads to filamentation and eventual cell lysis [5–7]. This was followed up later by deletion of the gene encoding PBP-3 resulting in ceftazidime resistance *in vitro* [8]. This alone, in the mode of action of ceftazidime is paradoxical, since cell division is an essential cellular process regardless of growth environment suggesting that an alternative encoded gene product can perform the function of the primary PBP-3 molecular target. In addition, resistance to ceftazidime *in vivo* has been associated with a single-nucleotide polymorphism (SNP) within the promoter region of the β-lactamase gene *penA* [9]. Together, these data indicate that the mode of action of ceftazidime and potential resistance mechanisms may well be different than its efficacious mode of action because of the dependence of the bacteria on the host growth environment.

Our group has also demonstrated conditional essentiality of a drug target *in vitro* versus *in vivo* [3]. This can be problematic when testing compound libraries through the conventional drug discovery pipelines. Novel molecular drug target identification against *B*. *pseudomallei* is also challenging due to the genome being comprised of two chromosomes and the resultant coding redundancy within the genome[10]. This leads to questioning essentiality of specific drug targets if multiple isoforms are encoded within the genome and observed differences in expression levels between isoforms under *in vitro* and *in vivo* growth environments. In order to better understand the efficacious mode of action, and the impact of the host disease environment on bacterial transcription and metabolism, and the essentiality of drug targets, mapping and comparing of the whole bacterial transcriptome in the host environment during drug treatment is necessary.

Accordingly, the transcriptional response of *B*. *pseudomallei* in the murine model of infection during ceftazidime treatment was compared to untreated and ceftazidime exposed *B*. *pseudomallei* growth under laboratory conditions using next generation sequencing. This is the first report of an *in vivo* whole bacterial transcriptional profiling from infected tissues and the unique transcriptional response of *B*. *pseudomallei* treated with ceftazidime in an animal model of infection, and resulting efficacious [*in vivo*] mode of action and transient resistance mechanisms. In addition, this analysis revealed bacterial molecular markers of treatment response. These studies are particularly important and promise to have an impact on rational targeted drug discovery for *B*. *pseudomallei* because it elucidates the transcriptionally active genes as well as potential drug resistance mechanisms. Further, the unique *in vivo* bacterial molecular markers revealed in this study promise to provide diagnostics to monitor the bacterial response to treatment.

## Materials and Methods

### Bacterial growth conditions and treatment *in vitro*

*B*. *pseudomallei* 1026b [11] was grown to an OD_600_ of ~0.6, frozen at -80°C in 10% glycerol and was used as the standard bacterial stock for these studies. For each evaluation bacteria were prepared fresh by growth from the standard stocks on Luria-Bertani (LB) Agar, Miller (BD) grown at 37°C for 48–72 h. Bacteria recovered from the LB plates were inoculated in 50 mL LB Broth. Broth cultures were then incubated for 18 h at 37°C passed 1:100 and incubated for an additional 6 h at 37°C. Cultures were then treated with 2X MIC (4μg/ml) ceftazidime or LB broth alone and incubated for an additional 2 h at 37°C. 1mL of each culture was centrifuged at 12,000rpm for 5 minutes, supernatant removed and bacteria resuspended in 1mL TRIzol (Invitrogen, Carlsbad, CA). Resuspended cultures were immediately stored at -80°C for further bacterial RNA isolation.

### Acute *B*. *pseudomallei* mouse model of infection and treatment

5–6 week old BALB/c female mice (Charles River Laboratories, Wilmington, MA) were challenged by intranasal infection with approximately 5,000 CFU/mouse *B*. *pseudomallei* 1026b[11] (N = 30 mice; 12 receiving treatment and 18 untreated). Animals were anesthetized with a mixture of 100 mg/kg ketamine (Aurora Veterinary Supply, Aurora, CO) and 10 mg/kg xylazine (Aurora Veterinary Supply) delivered intraperitoneally. The bacteria were diluted to the appropriate concentration in PBS to achieve an inoculum concentration of 2.5x10^5^ CFU/mL. The inoculum was then delivered in a 20 μL volume dropwise in alternating nostrils. Ceftazidime (Sigma Aldrich, St. Louis, MO) was formulated for injection in PBS (pH 7.4) at a concentration of 40 mg/mL and 200mg/kg was delivered inraperitoneally beginning at 36 hours post infection followed by a second dose at 48 hours post infection. Mice from the untreated group (Grp1) were euthanized at 36, 48, and 60 hours post infection and mice from the treated group (Grp2) sacrificed at 48 and 60 hours post infection. The number of viable bacteria in lung and spleen was determined for mice in each group at each timepoint (N = 9 Grp1;N = 6 Grp2) by plating serial 10-fold dilutions of homogenates onto LB agar and incubating for 48 h at 37^°^C. Lung and spleen were also homogenized in TRIzol from the remaining mice (N = 9 Grp1;N = 6 Grp2) and stored at -80°C for further bacterial RNA isolation.

### Isolation of bacterial transcripts

*In vitro* and infected mouse lung tissue samples were thawed and nucleic acid was isolated by organic partition. Samples were treated with DNAse (Fermentas, Burlington, Ontario) for 30 minutes and purified by phenol/chloroform/isoamyl alcohol (25:24:1) (Fisher Scientific, Pittsburgh, PA) extraction and ammonium acetate precipitation. Biological replicates (not pooled) were submitted to the CSU Next Generation Sequencing core for sample processing and sequencing. Briefly, RNA sample quality was determined on an Agilent 2100 Bioanylizer and samples with a RIN value greater than 8 passed the criteria for sequencing. Host transcripts were removed using MICROBEnrich (Life Technologies, Carlsbad, CA), sample libraries were prepared using the Ion Total RNA-Seq kit v2 (Life Technologies), and multiplexed on a P1 chip using Ionxpress RNA-Seq 1–16 kit (Life Technologies). Whole bacterial transcriptome sequencing was performed using the Ion Proton Next Generation Sequencer (Life Technologies).

### Analysis of next generation sequencing data

Data was received from the core in FASTQ format. Data files were uploaded to and analyzed using Galaxy [12–14]. FASTQ files were subjected to quality trimming by use of sickle with a minimum PHRED quality threshold greater than 20 and read length greater than 20bp. Trimmed reads from FASTQ files were aligned to *Burkholderia pseudomallei* 1026b genome (NCBI RefSeq NC_017831.1 & NC_017832.1) using Bowtie2 and gene expression determined using Cufflinks [15,16]. Expression output was normalized in FPKM format (fragments per kilobase of exon per million reads) [17,18]. Replicate mean values were calculated and data was further reduced to FPKM values greater than 2. The Accession number for the data in this study is PRJNA291046 and can be found at BioProject/NCBI under submission SUB863226.

### qRT-PCR validation

Three genes (BP1026B_II2144, BP1026B_II0025, and BP1026B_I0955) that were up or down regulated in both *in vitro* and *in vivo* ceftazidime treated conditions were selected and subjected to qRT-PCR to validate the normalized data generated from RNA-Seq and subsequent analysis. BP1026B_I3469 (16s rRNA) was used as a reference gene. Primer pairs used in qRT-PCR are listed in supplementary S1 Table cDNA was prepared from total RNA samples using Transcriptor First Strand cDNA Synthesis kit (Roche Applied Science). The resultant cDNA was used in downstream real-time PCR assays on a Roche LightCycler480. Samples were added to primer, LightCycler480 SYBR green I master mix (Roche), and water to final volume of 20μl. Real-time PCR cycle parameters were as follows: Pre-incubated at 95°C for 5 minutes, followed by 45 cycles of 95°C for 10 sec, 60°C for 10 sec, and 72°C for 10 sec. All biological replicate samples were quantified independently in technical triplicate.

### Ethics statement

All use of vertebrate animals at Colorado State University is conducted under AAALAC approval and has an OLAW number of A3572-01. Animals are housed in a state-of-the art ABL-3 facility that is supervised by full-time staff veterinarians and a large number of support staff. The CSU animal assurance welfare number is A3572-01 under file with the NIH. Veterinary care is consistent with the recommendations of the American Veterinary Medical Association (AVMA) Guidelines.

## Results

### Ceftazadime exposure and efficacious treatment in the acute model of disease

**Fig 1:**
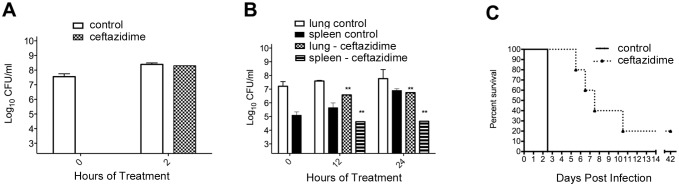
Ceftazidime treatment of B. pseudomallei 1026b *In vitro* and *In vivo*. (A) Mid-log phase *B*. *pseudomallei* 1026b was treated with 2X MIC (4μg/ml) for two hours and cells harvested for total RNA and CFU determination. (B) 5–6 week old Balb/c mice were infected with 5000 CFU *B*. *pseudomallei* 1026b. Mice were treated with 200 mg/kg ceftazidime intraperitoneally at 36 hours post infection and received a second dose at 48 hours post infection. Mice were euthanized and lungs harvested at 36, 48, and 60 hours post infection for total RNA and for CFU determination. Significance is determined by a p value <0.01 by Two-way ANOVA when compared to untreated control. (C) Ceftazidime treated mice were monitored for survival after withdrawal of treatment.

To assess concordance and unique differences in the transcriptional response of *in vivo* and *in vitro* sourced *B*. *pseudomallei* to ceftazidime treatment, bacteria obtained from infected tissues of infected animals treated with ceftazidime were compared to laboratory cultured and treated bacteria. As a comparative control mid-log cultures of *B*. *pseudomallei* 1026b were grown at 37°C under ambient conditions in rich medium and were exposed to 2X MIC ceftazidime for 2hrs. As indicated, there is no significant reduction in the number of viable bacteria after 2 hours of exposure to ceftazidime under laboratory conditions (Fig 1A). This drug exposure condition was chosen for this study because it is known from historical studies to result in a drug treatment specific transcriptional response before a more complex mixed response, which includes the cidal response [19–21].

The murine model of acute respiratory melioidosis was achieved by intranasal infection of Balb/c mice with ~5,000 CFU *B*. *pseudomallei* strain 1026b [4]. Treatment with 200mg/kg ceftazidime was delivered intraperitoneally at 36 and 48 hours post infection. Consistent with previous reports [4], respiratory infection of *B*. *pseudomallei* strain 1026b in the lungs grew rapidly resulting in a total lung burden of 7.2 Log_10_CFU/mL at 36 hours post infection (Fig 1B). After 48 hours of infection or at 12 hours of treatment there was a reduction in culturable bacteria in the lung of 1.02 Log_10_CFU/mL and 60 hours of infection and 24 hours of treatment there was a total bacterial reduction of 1.1 Log_10_CFU/mL. The bacterial burden in the spleens was also enumerated to monitor dissemination and treatment efficacy, which demonstrated that there was a significant reduction in the spleen as a result of treatment (Fig 1B). To further confirm that the infection and efficacious dose used in this *in vivo* study was consistent with previous studies, we monitored the untreated control group which succumbed to infection at 60 hours and the treatment group which had an observed 20–40% survival rate at day 42 (Fig 1C).

### Global transcriptional analysis of *B*. *pseudomallei* 1026b from *in vitro* and *in vivo* infected tissues

**Fig 2:**
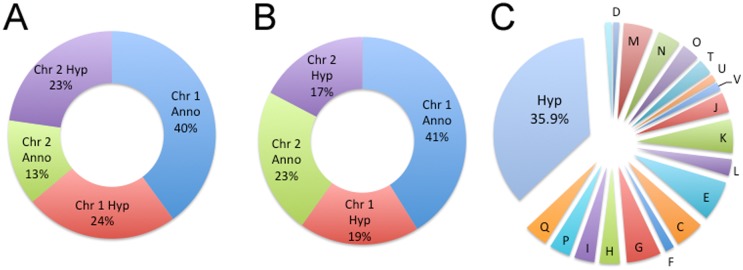
Global transcriptional response of *B*. *pseudomallei* 1026b. Distribution of *B*. *pseudomallei* 1026b transcripts for *in vitro* (A) and *in vivo* (B) grown samples. Chromosome 1 is approximately 4Mbp and encodes 3,724 genes while chromosome 2 is approximately 3.1 Mbp and encodes 2,538 genes for a total of 6,262 genes. Complete genomic profile was organized into COG (clusters of orthologous groups) categories and distribution plotted as a percentage of the total coding capacity 6,262 genes (C).

There is increasing need to understand how drugs with efficacy that are in clinical use exert a lethal effect on the bacteria and how the bacteria respond to exposure. To determine the differences between the effects of ceftazidime on *in vitro* laboratory grown bacteria and bacteria in infected tissues, we compared the global transcriptional response of *in vitro* grown *B*. *pseudomallei* 1026b and *B*. *pseudomallei* 1026b obtained from infected lungs in response to ceftazidime treatment using enrichment RNA-sequencing. The bacterial RNA from each sample was sequenced and the resulting FASTQ files were trimmed using sickle, aligned to the *B*. *pseudomallei* 1026b genome using Bowtie2, and cufflinks was used to determine gene expression. An average of 6.7 million reads were mapped per sample with each read length averaging 75bp. Whole genome mapping of the transcriptionally active ORFs identified from *in vitro* and *in vivo* grown bacteria revealed a fairly equal distribution between chromosome 1 or 2 regardless of origin with ratios of 64:36% and 60%:40%, respectively (Fig 2A and 2B). The global distribution of transcripts from these conditions does not support that there is a significant chromosome bias or preference for basic metabolism and adaptive responses as suggested previously [22]. The overall distribution of transcriptionally active ORFs was also assessed according to infection condition [*in vitro* versus *in vivo*] and by treatment and is shown in Table 1. To determine the common global metabolic activity of *in vitro* and *in vivo* grown bacteria, the transcriptionally active open reading frames were categorized into different metabolic function groups based on cluster of orthologous groups (COG) annotation assignments. This global analysis revealed that approximately 1/3 of the response fell into the category of unknown hypothetical while the next three categories most represented were amino acid transport and metabolism, carbohydrate transport and metabolism, and transcription (Fig 2C).

**Table 1:** Comparing global transcriptional response to treatment *in vitro* to treatment *in vivo*.

		*In vivo*			
	**# of Genes**	-	Unique to Untreated	Unique to Treatment	Common in Both
*In vitro*	-	1889	407	184	1504
	Unique to Untreated	94	112	63	336
	Unique to Treatment	74	85	46	166
	Common in Both	112	168	116	906

Total of 6,262 genes

**Fig 3:**
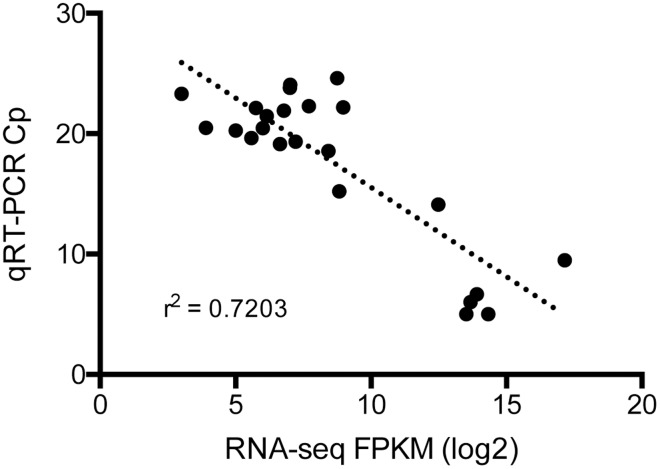
Correlation of RNA-seq and qRT-PCR. Fragments per kilobase of transcript per million mapped reads (FPKM) for differentially expressed genes and 16s reference gene were converted to Log2 values and plotted against respective qRT-PCR crossing point (Cp) values. Linear regression analysis (dotted line) was run to determine if the correlation coefficient was >0.7. An inverse relationship between Cp and FPKM is an indication of correlation and validation of the RNA-seq data set.

The RNA-Seq data was validated using qRT-PCR analysis of selected differentially expressed genes. The validation was performed comparing the crossing point (Cp) values to log_2_ FPKM values obtained from RNA-seq, which revealed an inverse relationship between the Cp and FPKM values. An inverse correlation between Cp and FPKM values for these genes is considered a standard validation of the RNA-seq data set [23]. This independent assessment revealed a strong concordance between the RNA-seq and quantitative PCR data (Fig 3).

### Transcriptional trends common to *in vitro* exposed *B*. *pseudomallei* and *B*. *pseudomallei* in the murine model of infection during ceftazidime treatment

**Fig 4:**
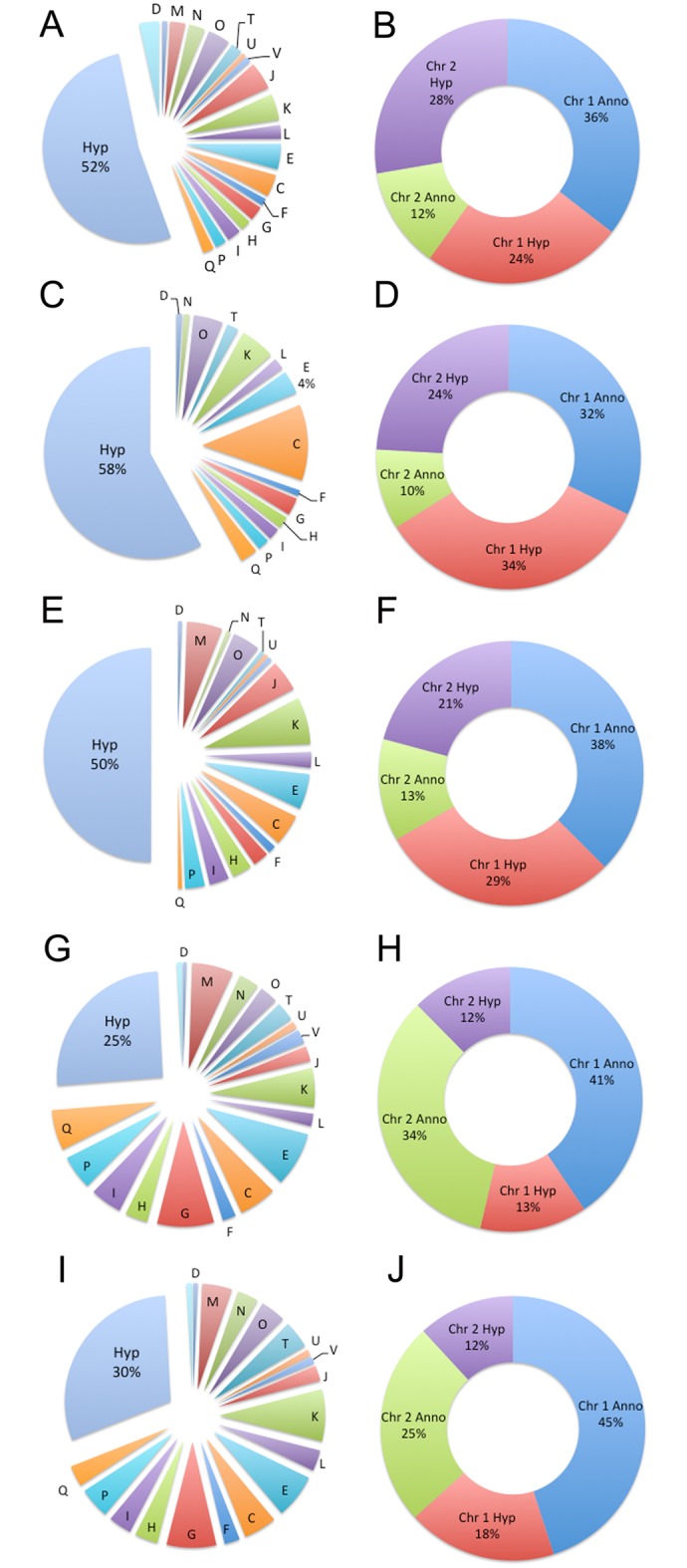
Transcriptionally active and differentially regulated genes in *B*. *pseudomallei* 1026b *in vivo*. Transcriptionally active open reading frames were categorized into different metabolic function groups based on cluster of orthologous groups (COG) annotation assignments and distribution of hypothetical and annotated genes indicated on each chromosome. Transcriptionally active genes during *in vitro* and *in vivo* treatment (**A**) and distribution of annotated and hypothetical genes encoded on each chromosome (**B**). Unique transcriptionally active genes *in vitro* (**C**) and distribution of annotated and hypothetical genes encoded on each chromosome (**D**). Differentially regulated genes during *in vitro* treatment (**E**) and distribution of annotated and hypothetical genes encoded on each chromosome (**F**). Unique transcriptionally active genes during *in vivo* growth (**G**) and distribution of annotated and hypothetical genes encoded on each chromosome (**H**). Differentially regulated genes during *in vivo* treatment (**I**) and distribution of annotated and hypothetical genes encoded on each chromosome (**J**). COG groups: D, cell division and chromosome partitioning; M, cell envelope biogenesis/outer membrane; N, cell motility and secretion; O, posttranslational modification/protein turnover/chaperons; T, signal transduction mechanisms; U, intracellular trafficking and secretion; V, defense mechanisms; J, translation/ribosomal structure and biogenesis; K, transcription; L, DNA replication/recombination/repair; E, amino acid transport and metabolism; C, energy production and conversion; F, nucleotide transport and metabolism; G, carbohydrate transport and metabolism; H, coenzyme metabolism; I, lipid metabolism; P, inorganic ion transport and metabolism; Q, secondary metabolites biosynthesis/transport and catabolism; Hyp, and poorly characterized.

A bacteria’s response to drug exposure and treatment is known to directly reflect the drugs mechanism of action [24]. However, the growth or environmental conditions during exposure and treatment also contribute and have a significant impact on the global transcriptional responses. As a result, the overlapping transcriptional responses from the direct activity of the drug and the environmental growth conditions can make it difficult to discern which responses to assign to the drugs mode of action and which responses to the environmental conditions. To identify the common bacterial response to ceftazidime the global *in vitro* bacterial transcriptional response to exposure to ceftazidime and the global *in vivo* bacterial transcriptional response during ceftazidime treatment in the acute model of melioidosis were subjected to concordance analysis to identify transcriptionally active features common to both *in vitro* and *in vivo* treated bacteria. This comparative analysis revealed 1,234 transcriptionally active open reading frames common to bacteria from *in vitro* conditions and from *in vivo* bacteria from infected tissues. Transcriptionally active ORFs or genes were defined as those present in all biological replicates for that condition and with an FPKM group mean greater than 2. This represents 19.7% of the total coding capacity of the *B*. *pseudomallei* 1026b genome. Genes annotated in known COG pathways during treatment *in vitro* and *in vivo* were more likely to be found on chromosome 1 at 36% of the total response compared to only 12% on chromosome 2. Unknown hypothetical transcriptionally active genes were found evenly distributed between the two chromosomes at 24% and 28%, respectively (Fig 4B).

Genes encoding unknown hypothetical proteins represented 52% of the transcriptionally active ORFs identified under this condition (Fig 4A), which is consistent with our observations in other studies and other reports of bacterial transcriptional responses to alternative conditions and stress [25]. While, the significance and biological role of genes encoding proteins with unknown function is difficult to define because of lack of annotation or biological information, genome context reveals some information. Examples of the most transcriptionally active ORFs in this category were BP1026B_I1021, BP1026B_I0763, and BP1026B_I0849. BP1026B_I1021 is a putative regulator of the *nar*-operon that encodes a respiratory nitrate reductase, which is known to be associated with denitrification and anaerobic nitrite respiration. BP1026B_I0763 encodes a hypothetical LysR-type transcriptional regulator family, which has been shown to be involved in regulation of diverse sets of genes involved in adaptive metabolism and virulence [26], and BP1026B_I0849 encodes a hypothetical protein containing the CreA regulatory domain, which is associated with a transcriptional regulator component of the regulatory domain controlling carbon source utilization [27].

COG groups for translation, ribosomal structure and biogenesis, and posttranslational modification, protein turnover, chaperones were the next groups most represented during treatment both *in vitro* and *in vivo*. Genes within the *rpl* operon, which encodes proteins of the large ribosomal subunit, and genes encoding tRNA synthases were represented in the ribosomal structure and biogenesis COG group. Heat shock proteins, *clp* and *hsl* proteases, and *dnaK* and *bicP* chaperones were among the most transcriptionally abundant within the COG category of posttranslational modification, protein turnover, and chaperones.

### Unique *B*. *pseudomallei* transcriptional response to *in vitro* growth

The number of transcriptionally active genes unique to *in vitro* growth, not differentially regulated during treatment, represents 112 genes or 2% of the coding capacity of *B*. *pseudomallei* and were mostly assigned to unknown hypothetical proteins (58% of 112) and energy production and conversion (12% of 112) (Fig 4C). Sixty percent of the genes that are uniquely expressed during *in vitro* growth mapped to chromosome 1 as compared to 40% to chromosome 2 (Fig 4D).

Of the ORFs categorized as hypothetical unknown function, BP1026B_I2494, BP1026B_I1886, and BP1026B_II2237 were the most transcriptionally active in this group. BP1026B_I2494 encodes a 119bp ncRNA that maps between a multidrug efflux transporter and unknown hypothetical protein, which the latter is suspected to play a role in regulation. BP1026B_I1886 encodes a 31aa protein that is located by the transcriptional regulator encoded by *osmT*, and segregation and condensation protein b, which is involved in chromosome separation. BP1026B_II2237 encodes a 203bp ncRNA flanked by a transposase and unknown hypothetical protein.

Genes most represented within the energy conservation and conversion COG group are part of the *nuo*-operon and genes encoding for ATP synthase subunits. Both are involved in energy generation. Genes in the *nuo*-operon encode the NADH dehydrogenase I, a key component of the respiratory chain, which is important for converting energy from reduced NADH. Coupled to the NADH dehydrogenase I process is the energy consuming process of ATP synthesis, which is consistent with the observed transcriptional activity of ATP synthase encoding genes. Together, the genes involved with this process are known to be essential for basic respiration and generation of energy in bacteria.

### Differentially expressed genes unique to *in vitro B*. *pseudomallei* ceftazidime treatment

There were 168 differentially regulated genes unique to *in vitro* treatment grouped in categories of unknown hypothetical (50% of the 168), transcription (7% of the 168), and energy production and conversion (5% of the 168) (Fig 4E). Differentially expressed genes unique to *in vitro* treatment are distributed more on chromosome 1 (66%) than on chromosome 2 (34%) (Fig 4F). Unknown hypothetical ORFs BP1026B_I1647, BP1026B_I1551, and BP1026B_II1658 were among the most transcriptionally active genes in untreated samples and ORFs BP1026B_I3414, BP1026B_II0244, and BP1026B_I3157 were the most active in treated samples *in vitro*. Many transcriptional regulators within the transcription COG category were differentially expressed during treatment *in vitro*. These included *tetR* that encodes an efflux regulator, *lysR*, which is associated with regulation of virulence, motility, and quorum sensing and the multidrug resistance regulator *marR*. The COG category energy production and conversion is the last major category that is differentially expressed during treatment *in vitro*. Most notable repressed in this group were the genes *cyoD*, which encodes the 12-kDa membrane protein ubiquinol oxidase subunit IV, and *ppa* encoding an inorganic pyrophosphatase which plays a role in lipid metabolism. Conversely, several genes in the succinate dehydrogenase (*sdh*) operon are induced during treatment.

### Identification of transient resistance mechanisms of *in vivo B*. *pseudomallei*

To understand how *B*. *pseudomallei* responds to treatment *in vivo* we first analyzed the metabolism unique to *in vivo* infection. Overall distribution of active ORFs or genes was more evenly assigned to COG pathways as compared to that observed *in vitro*. A total of 1,504 genes (24% coding capacity) were transcriptionally active and are unique to *in vivo* infection. The largest represented group was unknown hypothetical genes at 374 genes (25%). The next COG categories with the most transcriptionally active ORFs was carbohydrate transport and metabolism represented by 131 genes (9%), and amino acid transport and metabolism with 126 genes (8%) (Fig 4G).

The overall baseline transcriptional response unique to *in vivo* treatment was similarly distributed between the two chromosomes with 54% on chromosome 1 and 46% on chromosome 2 (Fig 4H). Carbohydrate transport and metabolism makes up the largest annotated class that is transcriptionally active during *in vivo* infection. Within this category several genes encoding ABC transporters and more specifically *araG* and *araH* of the L-arabinose transport operon. Five genes were transcriptionally active that are EmrB/QacA family drug resistance transporters and twenty-eight genes that encode membrane transport proteins in the major facilitator superfamily (MFS). Most transcriptionally active genes within this COG category were involved in more than one metabolic pathway within carbohydrate transport and metabolism. The top three transcriptionally active genes found were grouped in the hypothetical unknown category was BP1026B_II0381, BP1026B_II1410 (80bp ncRNA), and BP1026B_I1586 (164bp ncRNA). BP1026B_II0381 flanks 16s rRNA and another hypothetical unknown. BP1026B_II1410 flanks an encoded Lrp regulator, which is involved in putative regulation of amino acid metabolism and related genes. BP1026B_I1586 flanks a putative bacteriophage gp31, which encodes a protein analogous to chaperonin GroES.

Amino acid transport and metabolism COG category makes up the second largest annotated class that is transcriptionally active during *in vivo* infection. Within this category there are many operons that are important for basic metabolism *in vivo*. BP1026B_I0247-8 and BP1026B_II1815-7 are involved in tryptophan biosynthesis. BP1026B_I3366-7, *hisB* and *hisC* in this operon, are involved in histidine biosynthesis. BP1026B_II1824-6, *leuC* and *leuD* in the *leu*-operon, are involved in leucine biosynthesis. BP1026B_1410–11, the hom-1-thrC operon, are involved in threonine biosynthesis. Lastly, there are 23 genes, including *hisP*, *dppA*, *braC*, and *oppA*) that are classified as ABC transporters.

Notably, genes that encode penicillin-binding proteins (PBPs) and the penA β-lactamase were uniquely transcriptionally active *in vivo*. Altogether there were seven genes encoding PBPs that were transcriptionally active unique to *in vivo* infection one being BP1026B_II1292. The PBP 3 encoded by BP1026B_II1292 is known to be a target of ceftazidime and when harboring a deletion increases resistance *in vitro* [8]. The *penA* encoded β-lactamase also contributes to ceftazidime resistance and is uniquely expressed *in vivo*. This mechanism of ceftazidime resistance is attributed to an increase in expression due to point mutations caused by ceftazidime [28].

There were an additional 591 ORFs or 9.4% of the coding capacity identified as differentially expressed in the presence of ceftazidime in the *in vivo* infection. Similarly to the unique to *in vivo* conditions, the *in vivo* differentially regulated ORFs categorized dominantly to the COG categories of unknown hypotheticals, amino acid transport and metabolism, and carbohydrate transport and metabolism (Fig 4I). In addition to these COG groups, the COG group of transcription was also significantly represented at 8% total response. Transcriptional distribution within the genome shifted from a more balanced response observed during baseline metabolism *in vivo* to a more dominant response on chromosome 1 (63% of total) observed in differentially expressed genes in response to treatment *in vivo* (Fig 4J).

Unknown hypothetical genes were the most represented group within the differentially expressed genes *in vivo* at 178 or 30% of total. BP1026B_I2307, BP1026B_I0989, and BP1026B_I2303 were all the most transcriptionally active in untreated samples *in vivo* while BP1026B_I1657, BP1026B_II0389, and BP1026B_II1993 were most represented in the ceftazidime treated samples. All three encode putative proteins that ranged from 38 to 46aa in length. Both BP1026B_I2307 and BP1026B_I2303 flank 16s and 23s rRNA respectively. BP1026B_I0989 is downstream of a gene that encodes for the protein N-formylglutamate amidohydrolase. Only BP1026B_II0389 had a flanking annotated gene and that encodes a copper responsive transcriptional regulator.

Carbohydrate transport and metabolism was also uniquely represented with 45 genes or 0.7% of the coding capacity. Within this category we found seven genes differentially expressed that are ABC transporters, one gene that is a EmrB/QacA family drug resistance transporter, and twelve genes that encode membrane transport proteins in the MFS. BP1026B_I1984, a gene encoding beta-hexosaminidase, was found to be differentially expressed and is involved in beta-lactam resistance [29]. The *pgl-zwf* operon, involved in pentose phosphate metabolism and the *paa*-operon, involved in phenylacetic acid degradation were both differentially expressed and involved in carbohydrate metabolism.

Amino acid transport and metabolism and transcription both round out the top COG categories represented unique to *in vivo* treatment at 40 genes active per group. Many of the genes within the amino acid transport and metabolism category are involved in many different metabolic pathways within the category. Similar to what we had observed in bacteria grown under other conditions, ABC transporters were well represented within the data set with twelve genes differentially expressed. Out of the transcription COG category there were six different family transcriptional regulator types that were differentially expressed unique to *in vivo* treatment. Those six were AraC (15 total), LysR (14 total), GntR (3 total), lclR (3 total), and LuxR and PadR being represented by one gene respectively.

Importantly, the transcriptional response of *in vivo* bacteria in the presence of ceftazidime revealed the activity of genes that encode potential resistance mechanisms that are uniquely expressed *in vivo* upon ceftazidime treatment. The operon BP1026B *II2141-II2142-II2144-II2145* was identified to be differential expressed *in vivo* by ceftazidime treatment. This operon encodes a hypothetical protein of unknown function, a potential regulator in stress response and adaptation encoded by BP1026B_II2144, the DNA-binding response regulator *irlR2* (BP1026B_II2142) that has been associated with imipenem resistance through the regulation of OprD porin protein, which is involved in entry of carbapenem antibiotics, and BP1026B_II2145 that encodes a class D β-lactamase that is known to confer resistance to beta-lactam drugs through inactivation.

### Unique expression of pseudogenes under *in vivo* treatment conditions

The presence of pseudogenes and their role in bacterial physiology remains largely unknown. It is thought that translation of these genes that encode presumably non-functional proteins alter overall energy consumption, and their accumulation in bacterial genomes has been associated with pathogenesis within the host [30]. Analysis revealed that 0.4% of the genes (N = 9) that were unique to *in vivo* were pseudogenes, and these pseudogenes where encoded on chromosome 1 and chromosome 2 similar to expression found under other conditions. To substantiate the assignment of reads to these pseudogenes, BLAST analysis was performed. The reads uniquely mapped to these pseudogenes and did not map to homologous or paralogous gene sequences within the rest of the genome.

### Genome distributed molecular markers that are informative of ceftazidime treatment response

To identify genome distributed molecular markers of ceftazidime treatment response that can be used to assess whether bacteria are responding to treatment, bacterial molecular markers unique to *in vivo* infection and molecular markers specific to treatment response where sought. Molecular markers that map across the genome and include both chromosomes in the case of *B*. *pseudomallei* are preferable for monitoring viability and treatment response because as a group, their reporting potential is not as influenced by genome-transcriptional behavior. The full compliment of molecular markers that provide the foundation for a set of genome distributed molecular markers for monitoring treatment response consists of markers that indicate viability, are unique, and are responsive to treatment. Identification of bacterial molecular markers of *in vivo* infection serves as a positive control for viable infectious bacteria. Identification of bacterial molecular markers during in *in vivo* treatment distinguish if the bacteria are responding to treatment. To identify candidate molecular markers of *in vivo* infection, the transcriptional profile of bacteria unique to *in vivo* host growth conditions was analyzed to identify the bacterial molecular features that had the greatest expression as indicated by FPKM intensities. The features identified from this analysis where narrowed to the top 10 features. The top 10 most abundant molecular features group into three categories, genes that encode tRNA and rRNA, genes that encode proteins with unknown function and the *serS* seryl-tRNA synthetase (Table 2). Any of these molecular features are potential molecular markers for infection with *B*. *pseudomallei*.

**Table 2:** Molecular markers unique to *in vivo* infection.

Locus Tag	Gene Name	Product Name	COG	Untreated Mean FPKM	Ceftazidime Treated Mean FPKM
BP1026B_I0721	serS	seryl-tRNA synthetase	Translation, ribosomal structure and biogenesis	2300196	2948267
BP1026B_I0267		23S ribosomal RNA	rRNA/tRNA	134114	346519
BP1026B_I0264		tRNA-Ile	rRNA/tRNA	63341	70769
BP1026B_II0384		tRNA-Ala	rRNA/tRNA	39006	35264
BP1026B_I0263		16S ribosomal RNA	rRNA/tRNA	37367	25913
BP1026B_II0387		5S ribosomal RNA	rRNA/tRNA	30232	104503
BP1026B_II0383		tRNA-Ile	rRNA/tRNA	10674	13081
BP1026B_I3347		tRNA-Thr	rRNA/tRNA	8938	3590
BP1026B_II1410			Function unknown	7181	5317
BP1026B_I1586			Function unknown	4180	2709

**Fig 5:**
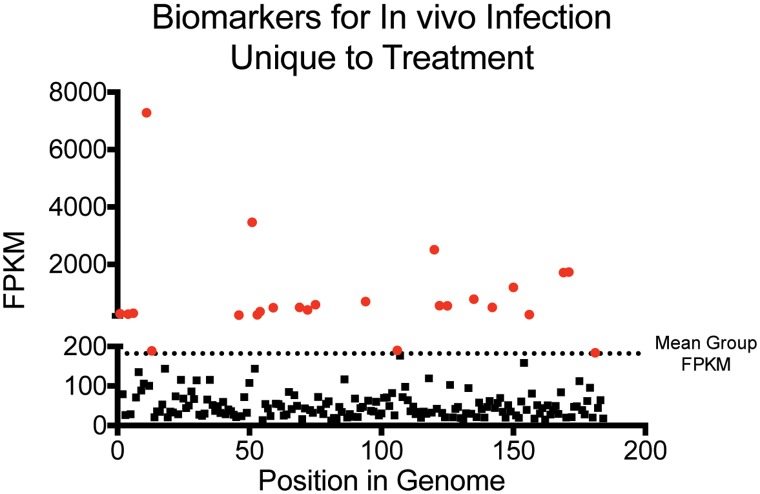
Biomarkers Unique to Ceftazidime Treatment *in vivo*. FPKM values from genes that were uniquely transcriptionally active *in vivo* during ceftazidime treatment were plotted on the y-axis and their position within the genome plotted on the x-axis. The mean FPKM was calculated from the treated *in vivo* transcript data set. Any gene with an FPKM value greater than that of the group mean was considered to b significant and is shown in red.

In addition to finding molecular markers that indicates a viable *B*. *pseudomallei* infection, a goal was to determine genome distributed molecular markers specific to ceftazidime susceptibility and treatment *in vivo* to inform treatment outcome (Fig 5). Accordingly we utilized the bacterial transcriptional profiles unique to *in vivo* treatment. This analysis identified 184 genes that were uniquely transcriptionally active during treatment *in vivo* with an average response of 182 FPKM, which is approximately 100 times the average abundance. The identified 182 gene features informative of *in vivo* treatment response were further enriched to the most abundant 25 (Table 3). These transcriptionally active genes were evenly distributed within the genome between both chromosomes and most are unknown hypotheticals (N = 14). Annotated genes of interest as potential biomarkers are *paaF* (BP1026B_I0261), *tagD-4* (BP1026B_II0586), and *filR* (BP1026B_I0034). Together, the molecular markers of *in vivo* infection and the molecular markers discriminant of ceftazidime treatment provide the foundation for diagnostics about response to treatment.

**Table 3:** Molecular markers ceftazidime treatment *in vivo*.

Locus Tag	Gene Name	Product Name	COG	Mean FPKM
BP1026B_I0261	paaF	enoyl-CoA hydratase	Lipid metabolism	7281
BP1026B_I1657		hypothetical protein	Function unknown	3468
BP1026B_II0389		hypothetical protein	Function unknown	2509
BP1026B_II2046		carboxylesterase family protein	Lipid metabolism	1729
BP1026B_II1993			Function unknown	1715
BP1026B_II1217		hypothetical protein	Function unknown	1197
BP1026B_II0985		hypothetical protein	Carbohydrate transport and metabolism	785
BP1026B_I3215		hypothetical protein	Function unknown	705
BP1026B_I2597		hypothetical protein	Function unknown	593
BP1026B_II0423		hypothetical protein	Function unknown	560
BP1026B_II0586	tagD-4	type VI secretion system	Cell motility and secretion	554
BP1026B_II1145		bacteriophage protein	gene/CDS	498
BP1026B_I2500		hypothetical protein	Function unknown	497
BP1026B_I2066		MerR family transcriptional regulator	Secondary metabolites biosynthesis, transport, and catabolism	490
BP1026B_I2534		hypothetical protein	Function unknown	411
BP1026B_I1865		hypothetical protein	Function unknown	349
BP1026B_I0239		hypothetical protein	Function unknown	297
BP1026B_I0156			Pseudogene	280
BP1026B_I0034	fliR	flagellar biosynthetic protein FliR	Cell motility and secretion	262
BP1026B_II1666			Function unknown	249
BP1026B_I1864		Flp pilus assembly protein, pilin Flp	Intracellular trafficking and secretion	239
BP1026B_I1508		TetR family transcriptional regulator	Cell envelope biogenesis, outer membrane	233
BP1026B_I3554	tag	DNA-3-methyladenine glycosylase I	DNA replication, recombination, and repair	190
BP1026B_I0335		serine-type carboxypeptidase family protein	Amino acid transport and metabolism	188
BP1026B_II2404		hypothetical protein	Function unknown	184

## Discussion

One of the challenging questions in disease treatment and management is, by what mechanism, as a consequence of inhibiting a molecular target, does a clinically used chemotherapeutic exert its lethal effect, and what are all the possible mechanisms of resistance, intrinsic, acquired or transient. It has been reported that the primary target of the clinically used drug, ceftazidime, is the PBP 3, FtsI protein involved in cell division [7]. Later studies demonstrated that PBP 3 could be knocked out indicating the presence of compensatory activity (6). In addition, *Burkholderia* spp. are known to be intrinsically resistant to the majority of clinically used chemotherapeutics [2]. Much work has been performed on the intrinsic resistance mechanisms of resistance in *Burkholderia* spp., for ceftazidime. However, this work has primarily focused on drug efflux and has been performed under *in vitro* laboratory conditions [2]. Only recently has the details of ceftazidime mode of action and resistance, as well as the role of the *penA* β-lactamase been studied [31]. While, these studies indicate that FtsI is a molecular target of ceftazidime, and ceftazidime is susceptible to PenA β-lactamase activity, additional factors may contribute to *in vivo* resistance to treatment. An approach to assessing the global metabolic activity of a bacterium under various growth conditions is *via* transcriptional profiling. Accordingly, we have used this approach to identify the conserved transcriptional response of *B*. *pseudomallei* during *in vivo* growth in the mouse model of infection and the unique *in vivo* transcriptional response to ceftazidime treatment.

Typically, a drug’s mode of action and resistance mechanisms are elucidated using *in vitro* studies, which are influenced by the artificial laboratory growth conditions under which they are performed [32]. We have observed that the coding capacity and coding redundancy of *B*. *pseudomallei* result in molecular drug targets that are conditionally bactericidal; specifically, some protein targets are only essential under specific growth conditions, which differ between *in vitro* and *in vivo* growth conditions [3]. Although informative, it may be difficult to determine a drugs efficacious mode of action and understand the mechanisms of drug resistance strictly from *in vitro* molecular studies that are routinely performed. Indeed, a pitfall with relying on *in vitro* only studies are that the molecular targets may not always be essential or even metabolically active *in vivo* as observed in this study, resulting in incomplete information or missed opportunities.

The *in vivo* transcriptional response provides a starting point to better understand the functional association of unknown hypothetical proteins that are expressed during infection and differentially expressed during treatment. To get a better understanding on how the molecular target profiles differ *in vitro* compared to *in vivo* and potential alternative metabolic pathways that may influence susceptibility to ceftazidime we sequenced the transcriptome of *B*. *pseudomallei* from *in vitro* cultures and *in vivo* infected tissue in the presence of ceftazidime. The transcriptional profiles were compared to determine differences in transcriptional diversity, utilization of coding capacity and protein homolog usage, as a surrogate for assessing the impact on metabolism. The most significant differences observed between *in vitro* grown bacteria and bacteria in infected tissues were observed in the transcription of hypothetical genes, genes encoding energy production, carbohydrate, amino acid and lipid metabolism, and secondary metabolites. This is attributed to the controlled enriched nutritional and environmental conditions of *in vitro* laboratory culturing that allow the bacteria to use only a minimal number of metabolic pathways. Similarly, there was greater bacterial transcriptional diversity during treatment *in vivo* compared to treatment *in vitro*; the number of significantly differentially expressed genes *in vivo* outnumbered those observed *in vitro* ~2:1. There was ~20% reduction in the number of transcriptionally active genes that encode hypothetical genes. Genes that encode proteins involved in alternative transcriptional regulation, proteome remodeling and protein turnover and secretion where differentially regulated in *in vivo* bacteria as compared to *in vitro* grown and treated bacteria. The significant difference in transcriptional activity in hypothetical genes represented approximately 33% of the total data set indicating the use and importance of the coding capacity that provides critical evidence to better understanding how *B*. *pseudomallei* responds during treatment (under stress) in the host and the potential for discovery of novel molecular targets and molecular markers.

The transcriptional response of *in vivo* bacteria revealed the activity of genes that encode potential transient resistance mechanisms to ceftazidime. The operon BP1026B *II2141-II2142-II2144-II2145* operon that encodes a regulator, an OprD porin protein associated with imipenem resistance and a class D β-lactamase (known to confer resistance to β-lactam drugs through inactivation), demonstrates that significant differences in bacterial transcriptional activity occur *in vivo* that may have a significant impact on susceptibility to treatment and outcome. In addition to the PBP BP1026B_II1292 identified as a target for ceftazidime treatment [8], there are six others that are uniquely transcriptionally active *in vivo*. These other PBPs could serve as potential non-susceptible targets that in fact sequester drug decreasing its effectiveness. Interestingly, these multiple PBP targets are only transcriptionally active *in vivo* suggesting their effect on drug sequestration wouldn’t be evident when testing *in vitro*. The identification of this operon as well as several PBPs and a β-lactamase that are uniquely active *in vivo* substantiates that there maybe potential transient resistance mechanisms uniquely active during infection that would not be identified by *in vitro* studies.

Interestingly, genes that encode pseudogenes were also identified. The transcriptional activity of genes that encode non-functional proteins can result from transcriptional coupling as part of a polycistron or transcribed from their own promoter. In this instance, the majority of the transcriptionally active pseudogenes identified is located in regions of the chromosome with greater number of genes encoding hypothetical unknown proteins, and are flanked by hypothetical genes. This raises the possibility that pseudogenes where once hypothetical genes that underwent evolutionary decay. While the potential impact of pseudogenes on bacterial metabolism is unclear, there expression in *B*. *pseudomallei* is consistent with reports that demonstrate other bacterial pathogens express genes encoding non-functional proteins in *in vivo* environments [30].

Disease treatment and outcome monitoring is becoming increasing important with the emergence of drug resistance. In fact, disease management in a clinical setting would be improved if treatments were accompanied with early indication diagnostics of treatment outcome. This is particularly true for emerging pathogens such as *B*, *pseudomallei* that cause fatal acute infections and that are naturally resistant to treatment because of the short opportunity for effective treatment. In addition, the ability of *B*. *pseudomallei* to establish a chronic infection complicates disease management. Therefore, there is a need for diagnostic molecular markers capable of monitoring disease progression and treatment outcome, and to report the activity of potential resistance mechanisms. The current methodologies for detection of *Burkholderia pseudomallei* infection relay heavily on serological tests and PCR assays that are low in sensitivity and specificity of detection [33]. There is growing interest in using RNAseq data sets as tools in discovery of new biomarkers that are relevant to host-pathogen interaction and response to treatment [34].

These data underscore the importance to study the bacterial response to drug treatment in *in vivo* conditions. It is clear that the growth environment and nutrient conditions have a profound impact on the bacterial response and may influence to a great degree the susceptibly to treatment. Molecular target availability *in vivo* is essential to assess efficacy of current clinically used drugs and compounds progressing through drug development. Researchers could be missing out on many potential drug candidates due to the limited transcriptional diversity observed *in vitro*. Further, this report highlights the potential of transient or phenotypic resistance mechanisms, and provides a better understanding of how the bacteria respond to treatment *in vivo*, which may account for the observed differences between bacterial treatment response *in vitro* versus *in vivo*.

## Supporting Information

S1 Table (DOCX)

## References

[1] Schiebel J, Chang A, Shah S, Lu Y, Liu L, Pan P, et al Rational Design of Broad Spectrum Antibacterial Activity Based on a Clinically Relevant Enoyl-Acyl Carrier Protein (ACP) Reductase Inhibitor. J Biol Chem. 2014;289: 15987–16005. 10.1074/jbc.M113.532804 24739388 PMC4047375

[2] Schweizer HP. Mechanisms of antibiotic resistance in Burkholderia pseudomallei: implications for treatment of melioidosis. Future Microbiol. 2012;7: 1389–1399. 10.2217/fmb.12.116 23231488 PMC3568953

[3] Cummings JE, Kingry LC, Rholl DA, Schweizer HP, Tonge PJ, Slayden RA. The Burkholderia pseudomallei enoyl-acyl carrier protein reductase FabI1 is essential for in vivo growth and is the target of a novel chemotherapeutic with efficacy. Antimicrob Agents Chemother. 2014;58: 931–935. 10.1128/AAC.00176-13 24277048 PMC3910854

[4] Cummings JE, Beaupre AJ, Knudson SE, Liu N, Yu W, Neckles C, et al Substituted diphenyl ethers as a novel chemotherapeutic platform against Burkholderia pseudomallei. Antimicrob Agents Chemother. 2014;58: 1646–1651. 10.1128/AAC.02296-13 24379198 PMC3957837

[5] Spratt BG. Distinct penicillin binding proteins involved in the division, elongation, and shape of Escherichia coli K12. Proc Natl Acad Sci U S A. 1975;72: 2999–3003. 1103132 10.1073/pnas.72.8.2999 PMC432906

[6] Hayes MV, Orr DC. Mode of action of ceftazidime: affinity for the penicillin-binding proteins of Escherichia coli K12, Pseudomonas aeruginosa and Staphylococcus aureus. J Antimicrob Chemother. 1983;12: 119–126. 6413485 10.1093/jac/12.2.119

[7] Tavío MM, Aquili VD, Vila J, Poveda JB. Resistance to ceftazidime in Escherichia coli associated with AcrR, MarR and PBP3 mutations and overexpression of sdiA. J Med Microbiol. 2014;63: 56–65. 10.1099/jmm.0.063727-0 24089577

[8] Chantratita N, Rholl DA, Sim B, Wuthiekanun V, Limmathurotsakul D, Amornchai P, et al Antimicrobial resistance to ceftazidime involving loss of penicillin-binding protein 3 in Burkholderia pseudomallei. Proc Natl Acad Sci U S A. 2011;108: 17165–17170. 10.1073/pnas.1111020108 21969582 PMC3193241

[9] Sarovich DS, Price EP, Limmathurotsakul D, Cook JM, Von Schulze AT, Wolken SR, et al Development of ceftazidime resistance in an acute Burkholderia pseudomallei infection. Infect Drug Resist. 2012;5: 129–132. 10.2147/IDR.S35529 22977307 PMC3430440

[10] Holden MTG, Titball RW, Peacock SJ, Cerdeño-Tárraga AM, Atkins T, Crossman LC, et al Genomic plasticity of the causative agent of melioidosis, Burkholderia pseudomallei. Proc Natl Acad Sci U S A. 2004;101: 14240–14245. 10.1073/pnas.0403302101 15377794 PMC521101

[11] DeShazer D, Brett PJ, Carlyon R, Woods DE. Mutagenesis of Burkholderia pseudomallei with Tn5-OT182: isolation of motility mutants and molecular characterization of the flagellin structural gene. J Bacteriol. 1997;179: 2116–2125. 9079894 10.1128/jb.179.7.2116-2125.1997 PMC178945

[12] Giardine B, Riemer C, Hardison RC, Burhans R, Elnitski L, Shah P, et al Galaxy: a platform for interactive large-scale genome analysis. Genome Res. 2005;15: 1451–1455. 10.1101/gr.4086505 16169926 PMC1240089

[13] Blankenberg D, Von Kuster G, Coraor N, Ananda G, Lazarus R, Mangan M, et al Galaxy: a web-based genome analysis tool for experimentalists. Curr Protoc Mol Biol Ed Frederick M Ausubel Al. 2010;Chapter 19: Unit 19.10.1–21. 10.1002/0471142727.mb1910s89 PMC4264107 20069535

[14] Goecks J, Nekrutenko A, Taylor J, Galaxy Team. Galaxy: a comprehensive approach for supporting accessible, reproducible, and transparent computational research in the life sciences. Genome Biol. 2010;11: R86 10.1186/gb-2010-11-8-r86 20738864 PMC2945788

[15] Langmead B, Salzberg SL. Fast gapped-read alignment with Bowtie 2. Nat Methods. 2012;9: 357–359. 10.1038/nmeth.1923 22388286 PMC3322381

[16] Trapnell C, Williams BA, Pertea G, Mortazavi A, Kwan G, van Baren MJ, et al Transcript assembly and quantification by RNA-Seq reveals unannotated transcripts and isoform switching during cell differentiation. Nat Biotechnol. 2010;28: 511–515. 10.1038/nbt.1621 20436464 PMC3146043

[17] Mortazavi A, Williams BA, McCue K, Schaeffer L, Wold B. Mapping and quantifying mammalian transcriptomes by RNA-Seq. Nat Methods. 2008;5: 621–628. 10.1038/nmeth.1226 18516045 PMC13303166

[18] Dillies M-A, Rau A, Aubert J, Hennequet-Antier C, Jeanmougin M, Servant N, et al A comprehensive evaluation of normalization methods for Illumina high-throughput RNA sequencing data analysis. Brief Bioinform. 2013;14: 671–683. 10.1093/bib/bbs046 22988256

[19] Sullivan TJ, Truglio JJ, Boyne ME, Novichenok P, Zhang X, Stratton CF, et al High affinity InhA inhibitors with activity against drug-resistant strains of Mycobacterium tuberculosis. ACS Chem Biol. 2006;1: 43–53. 10.1021/cb0500042 17163639

[20] Boyne ME, Sullivan TJ, amEnde CW, Lu H, Gruppo V, Heaslip D, et al Targeting fatty acid biosynthesis for the development of novel chemotherapeutics against Mycobacterium tuberculosis: evaluation of A-ring-modified diphenyl ethers as high-affinity InhA inhibitors. Antimicrob Agents Chemother. 2007;51: 3562–3567. 10.1128/AAC.00383-07 17664324 PMC2043287

[21] England K, am Ende C, Lu H, Sullivan TJ, Marlenee NL, Bowen RA, et al Substituted diphenyl ethers as a broad-spectrum platform for the development of chemotherapeutics for the treatment of tularaemia. J Antimicrob Chemother. 2009;64: 1052–1061. 10.1093/jac/dkp307 19734171 PMC2760461

[22] Cooper VS, Vohr SH, Wrocklage SC, Hatcher PJ. Why genes evolve faster on secondary chromosomes in bacteria. PLoS Comput Biol. 2010;6: e1000732 10.1371/journal.pcbi.1000732 20369015 PMC2848543

[23] Wang Y, Li X, Mao Y, Blaschek HP. Single-nucleotide resolution analysis of the transcriptome structure of Clostridium beijerinckii NCIMB 8052 using RNA-Seq. BMC Genomics. 2011;12. 21962126 10.1186/1471-2164-12-479 PMC3271303

[24] Kohanski MA, Dwyer DJ, Collins JJ. How antibiotics kill bacteria: from targets to networks. Nat Rev Microbiol. 2010;8: 423–435. 10.1038/nrmicro2333 20440275 PMC2896384

[25] Galperin MY. “Conserved hypothetical” proteins: prioritization of targets for experimental study. Nucleic Acids Res. 2004;32: 5452–5463. 10.1093/nar/gkh885 15479782 PMC524295

[26] Maddocks SE, Oyston PCF. Structure and function of the LysR-type transcriptional regulator (LTTR) family proteins. Microbiol Read Engl. 2008;154: 3609–3623. 10.1099/mic.0.2008/022772-0 19047729

[27] Ries LNA, Beattie SR, Espeso EA, Cramer RA, Goldman GH. Diverse Regulation of the CreA Carbon Catabolite Repressor in Aspergillus nidulans. Genetics. 2016;203: 335–352. 10.1534/genetics.116.187872 27017621 PMC4858783

[28] Randall LB, Dobos K, Papp-Wallace KM, Bonomo RA, Schweizer HP. Membrane-Bound PenA β-Lactamase of Burkholderia pseudomallei. Antimicrob Agents Chemother. 2016;60: 1509–1514. 10.1128/AAC.02444-15 PMC4775993 26711764

[29] Zeng X, Lin J. Beta-lactamase induction and cell wall metabolism in Gram-negative bacteria. Front Microbiol. 2013;4. 10.3389/fmicb.2013.00128 PMC3660660 23734147

[30] Williams DL, Slayden RA, Amin A, Martinez AN, Pittman TL, Mira A, et al Implications of high level pseudogene transcription in Mycobacterium leprae. BMC Genomics. 2009;10: 397 10.1186/1471-2164-10-397 19706172 PMC2753549

[31] Rholl DA, Papp-Wallace KM, Tomaras AP, Vasil ML, Bonomo RA, Schweizer HP. Molecular Investigations of PenA-mediated β-lactam Resistance in Burkholderia pseudomallei. Front Microbiol. 2011;2. 10.3389/fmicb.2011.00139 PMC3129521 21747814

[32] Brown Michael. Influence of Growth Rate on Susceptibility to Antimicrobial Agents: Modification of the Cell Envelope and Batch and Continuous Culture Studies. Antimicrob Agents Chemother. 1990;34: 1623–1628. 2285273 10.1128/aac.34.9.1623 PMC171894

[33] Lau SKP, Sridhar S, Ho C-C, Chow W-N, Lee K-C, Lam C-W, et al Laboratory diagnosis of melioidosis: past, present and future. Exp Biol Med Maywood NJ. 2015;240: 742–751. 10.1177/1535370215583801 PMC4935216 25908634

[34] Dix A, Vlaic S, Guthke R, Linde J. Use of systems biology to decipher host-pathogen interaction networks and predict biomarkers. Clin Microbiol Infect Off Publ Eur Soc Clin Microbiol Infect Dis. 2016; 10.1016/j.cmi.2016.04.014 27113568

