# Supplementary material for: Transient In Vivo Resistance Mechanisms of Burkholderia pseudomallei to Ceftazidime and Molecular Markers for Monitoring Treatment Response
Source: PLoS Negl Trop Dis. 2017 Jan 12;11(1):e0005209. doi: 10.1371/journal.pntd.0005209 (PMC5230754; doi:10.1371/journal.pntd.0005209)
Supplement: S1 Table — (DOCX) [file pntd.0005209.s001.docx]

**Table S1 Primers used in this study for quantitative PCR**

| Gene | Forward Primer 5’-3’ | Reverse Primer 5’-3’ |
| --- | --- | --- |
| BP1026B_I3469 | AAATGCGTAGAGATGTGGAGG | TATCTAATCCTGTTTGCTCCCC |
| BP1026B_II0025 | GTGGACGCGAAAAAGATCA | TCATCGTGATCGTCAGCTTG |
| BP1206B_II2144 | GCCACATTCCTCACTGTCG | CGACCAGCTCCTGATAGACC |
| BP1026B_I0995 | CGAATTTCCGGAACGAGAG | CATCAACGCTGTACACGACA |
